# Sesamin from *Cuscuta palaestina* natural plant extracts: Directions for new prospective applications

**DOI:** 10.1371/journal.pone.0195707

**Published:** 2018-04-10

**Authors:** Saleh Abu-Lafi, Sadam Makhamra, Ibrahim Rayan, Waseim Barriah, Ahmed Nasser, Basheer Abu Farkh, Anwar Rayan

**Affiliations:** 1 Faculty of Pharmacy, Al-Quds University, Abu-Dies, Palestine; 2 Chemistry Department, Faculty of Science and Technology, Al-Quds University, Abu-Dies, Palestine; 3 Institute of Applied Research—Galilee Society, Shefa-Amr, Israel; 4 Al-Qasemi College of Engineering & Science, Baka EL-Garbiah, Israel; 5 The Interinstitutional Analytical Instrumentation Unit, the Volcani Center, ARO, Rishon LeZion, Israel; 6 Drug Discovery Informatics Lab, QRC—Qasemi Research Center, Al-Qasemi Academic College, Baka El-Garbiah, Israel; King Saud University College of Pharmacy, SAUDI ARABIA

## Abstract

The aim of this study is to disclose the potential bioactive components of *Cuscuta palaestina*, a native parasitic natural plant of flora palaestina and to open direction towards new prospective application. GC-MS analysis identified 18 components in the methanolic extract of *C*. *palaestina* for the first time. The most appealing among them are Sesamin and two other phytosterols (Campesterol and Stigmasterol), all of which are documented in the scientific literature for their anticancer activity. Quantitation of Sesamin extracted from *C*. *palaestina* by HPLC-PDA with the use of three organic solvents showed that the Sesamin content in the methanolic extract was the highest. Following the disclosure of Sesamin presence in *C*. *palaestina*, we raised the question of whether it is produced naturally in *C*. *palaestina* or acquired from the host plant. The quantitation of Sesamin in *C*. *palaestina* was performed while being with five different host plants, and was compared with the amount of Sesamin in *C*. *palaestina* grown alone. The findings reveal that Sesamin is an endogenous secondary metabolite in *C*. *palaestina*. Thus, further studies are required to prove if *C*. *palaestina* can be used as an alternative source of anticancer phytochemicals, mainly Sesamin, and if proteins in the Sesamin production pathway could be valid biological targets for the development of novel and selective pesticides for control/ eradication of *C*. *palaestina* and maybe some other *Cuscuta* species. As well, the findings from this study raise a big question of whether inferring Sesamin production in *C*. *palaestina* could reduce its attack ability to host plants.

## Introduction

Recent years have witnessed a renewed interest in plants as an alternative avenue to the discovery of new pharmaceuticals. This interest is driven by both academia and the pharmaceutical industry and has led to the espousal of crude extracts of plants for self-medication by the public. Plants used in traditional medicine, therefore, have an increasingly important role to play in the maintenance of health worldwide and play an important role in the introduction of new treatments. The World Health Organization (WHO) estimates that up to eighty percent of the world’s population relies on traditional medicine for some aspect of primary health care. [1] Herbal medicines have been universally accepted and have had a great impact on the world of health and international trade. More than 60 percent of the anticancer chemotherapeutic drugs in current use have been derived in one way or another from natural sources, including plants. We can identify potential bioactive compounds via a bio-guided fractionation process [2, 3] or with *in silico* techniques, [4–10] followed by *in vitro/in vivo* experiments. The estimated number of plant species on earth ranges between 500,000 and 1,250,000, and less than ten percent of these have been studied chemically and pharmacologically for their potential medicinal value. [11]

*C*. *palaestina*, which belongs to the family *Convolvulaceae*, is an extensive climber parasite. It is more often called *dodder* in English and *Halook* or *Hamool Falastini* in Arabic. The mature plants have no connection to the ground. The plant has no chlorophyll and cannot make its own nutrients by photosynthesis. The stem consists of thread-like filaments that grow and attach themselves to nearby host plants. *Cuscuta* is a genus of about 100–170 species. [12] There are no data in the literature concerning the analysis of anti-proliferative and anticancer activity in these species nor the chemical composition of their constituents. As part of our efforts to find potential sources of agents that inhibit cancer development, we have investigated the anticancer effects of *C*. *palaestina* crude extract on the colon carcinoma cell line. According to an intensive literature survey, no GC-MS method has yet been reported for the determination of the phytochemicals present in *C*. *palaestina*. The main constituents of the methanol and hexane extracts from this plant were explored and investigated by GC-MS in the electron impact mode and are reported in this paper for the first time. Moreover, an analytical method was designed to quantify the Sesamin amount present in the plant by using HPLC-PDA. The results show that *C*. *palaestina* provides a reliable and enriched alternative source of Sesamin, which is commonly isolated from sesame seeds.

## Material and methods

### Plant collection and extract preparation

Samples of entire *C*. *palaestina* plants, comprising the stems and flowers, as well as their host plants, were collected from Kabul fields (near Acre). There is no specific permissions were required for the used locations/activities and the field studies did not involve endangered or protected species. The plant was washed with distilled water, and dried in the shade. Quantities of fifty milliliters of water and five different organic solvents (hexane, methanol, ethanol, ethyl acetate and chloroform) were added to the dried ground plant material (5 g) in a beaker, and the samples were sonicated for 120 min at 45°C and then left for 4 h to complete extraction. Samples of 25 milliliters from all the extracts were concentrated with a rotary vacuum evaporator under reduced pressure to determine yields and concentrations, and the rest of the extract was used for GC-MS studies and for quantification by HPLC.

### GC-MS analysis condition

Components of *C*. *palaestina* from the methanol and hexane extracts were run and identified using a GC-MS system (Agilent Technologies 7890A) coupled with a mass spectrometer (Agilent Technologies 5975C, inert MSD with a triple-axis detector). The GC was operated on an Agilent J&W GC column HP-5 column (30 m x 0.32 mm, id. with a 0.25-μm film thickness). The carrier gas was helium, at a flow rate of 1.2 mL/min, and the injection volume was 1μL. The injection port temperature was 300°C, and the ionization voltage was 70eV. The samples were injected in split mode with a ratio of 10:1. Mass spectra were recorded with a scan every second over a range of 45–800 (*m/z*). The oven temperature program was started at 50°C and set for 5 min, ramped up to 320°C with a heating rate of 5°C/min, and then finally held for an extra 20 min. The injection port temperature was 280°C, and the MS interface temperature was 300°C. The solvent delay time was 7 min, in order to get rid of the gigantic solvent peak. The mass spectra obtained were preliminarily interpreted by comparing them with data in the Mass Spectral Library of the National Institute of Standards and Technology (NIST, Gaithersburg, USA).

### Method for the quantitative analysis of the Sesamin content of C. palaestina by analytical HPLC-PDA

Chromatographic conditions were used to separate and quantify the Sesamin in *C*. *palaestina*. Crude samples were run on a reversed-phase ODS column by Waters (XBridge, 4.6 ID x 150 mm, 5 μm), with a guard column of XBridge ODS (20 mm x 4.6mm ID, 5 μm). The mobile phase consisted of binary solvent mixture of 0.5% acetic acid solution (A) and acetonitrile (B), in linear gradient mode. The start was a 100% (A) that descended to 70% (A) in 40 min. Then to 40% (A) in 20 min and finally to 10% (A) in 2 min and stayed there for 6 min and then back to the initial conditions in 2 min. The HPLC system was equilibrated for 5 min with the initial acidic water mobile phase (100% A) before the next sample was injected. All of the samples were filtered with a 0.45-μm PTFE filter. The PDA wavelengths ranged from 210–500 nm, the flow rate was 1 mL/min, the injection volume was 10 μl, and the column temperature was room temperature. The Sesamin was eluted with a retention time of 54.6 min.

### Calibration curve of the standards

**Fig 1:**
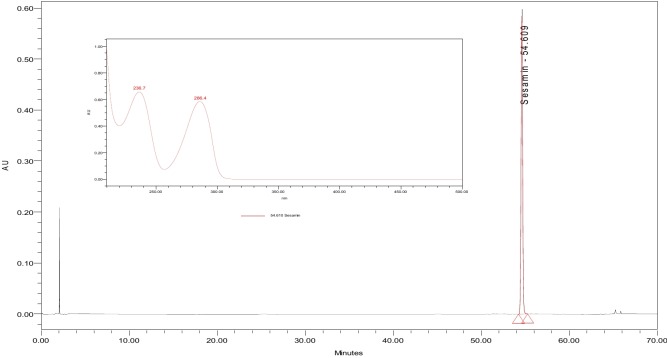
Typical analytical HPLC-PDA chromatogram of standard Sesamin at concentration of 250 ppm; the UV-Vis spectrum maxima is at a λ of 235.5 and 286.4 nm.

Sesamin stock solution was prepared by dissolving 10 mg of Sesamin reference standard (purchased from Sigma Aldrich, Israel) in 40 mL ethanol, which produced a concentration of 250 ppm. The solution was filtered using a 0.45-μm membrane. A five-point calibration curve was constructed by injecting the diluted solution into the HPLC-PDA, and the value of the coefficient of determination (R^2^) was > 0.999. Fig 1 displays a typical HPLC-PDA chromatogram of the Sesamin that was used in the current study.

## Results and discussion

**Fig 2:**
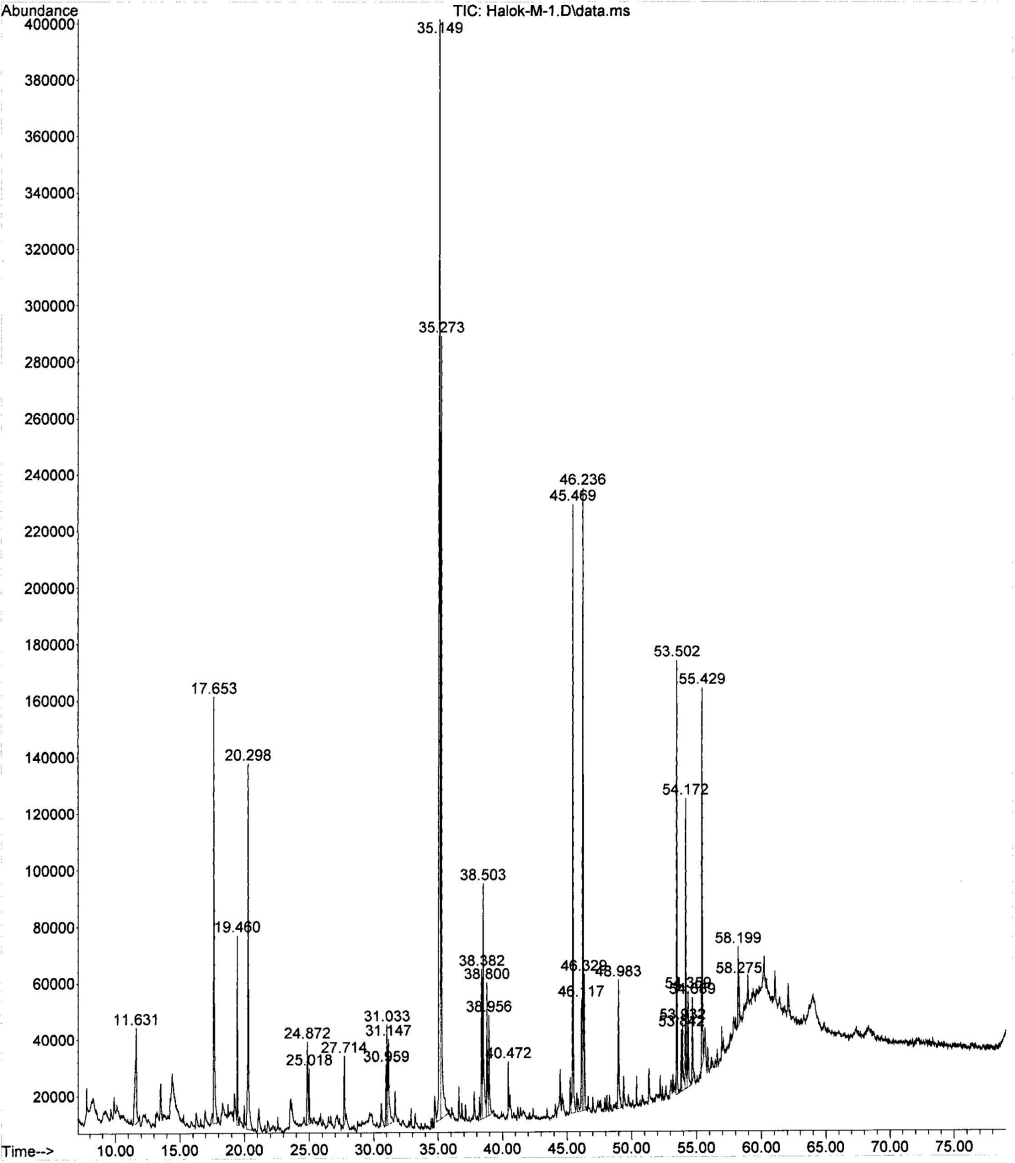
GC-MS analysis of the methanolic extract of *C*. *palaestina*.

**Fig 3:**
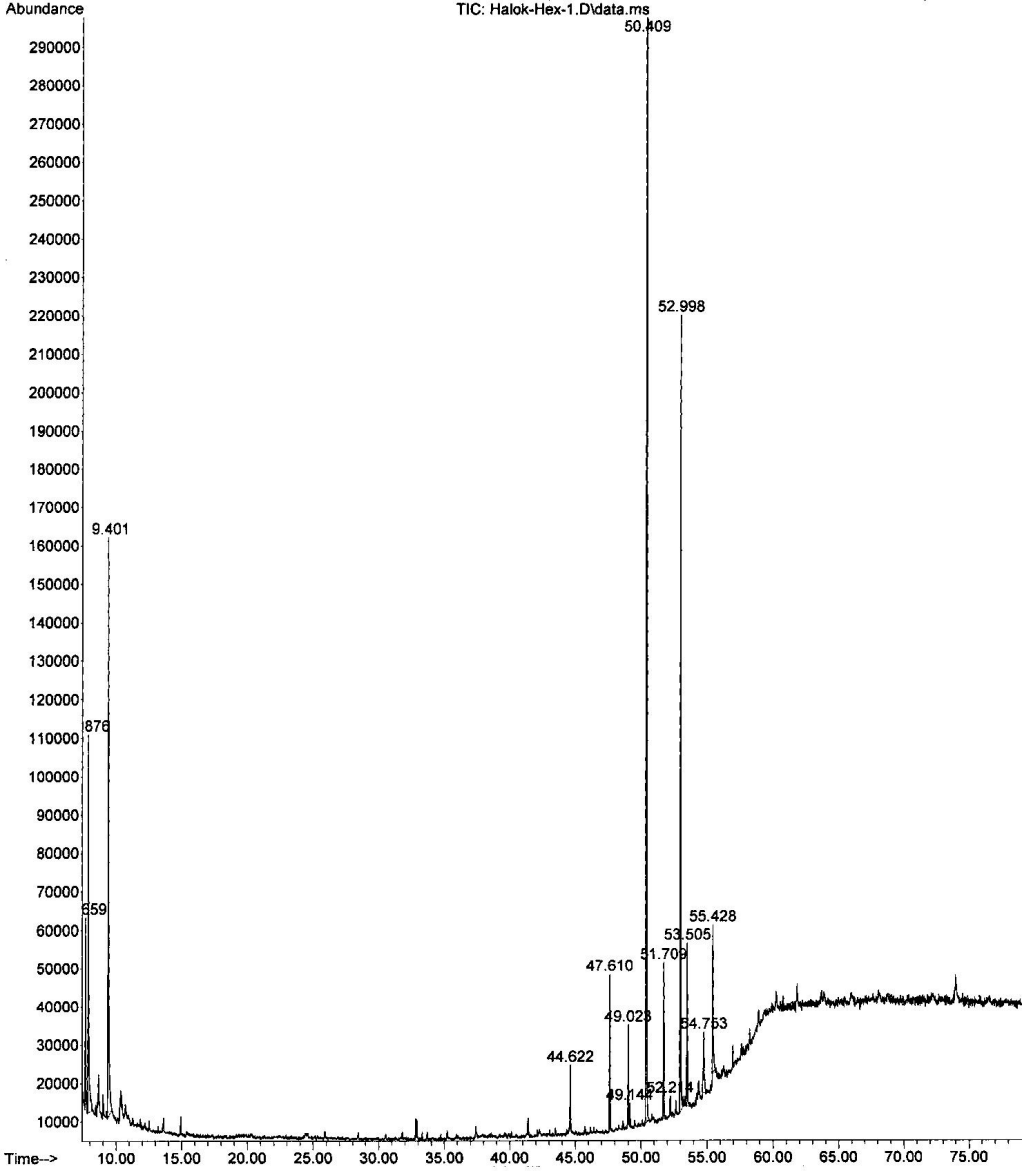
GC-MS analysis of the hexane extract of *C*. *palaestina*.

**Fig 4:**
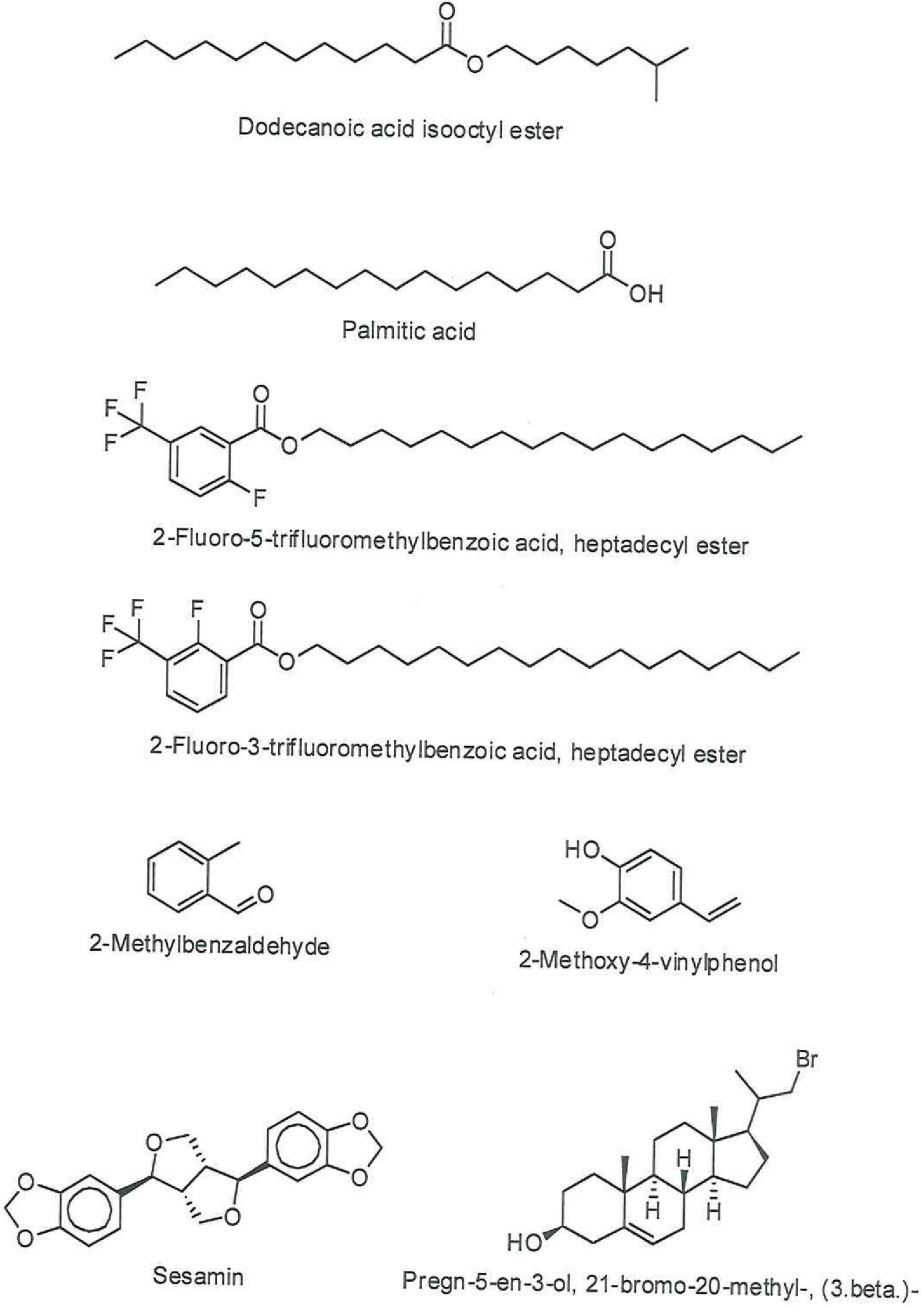
Chemical structures of the major components in the methanolic *C*. *palaestina* extract.

**Fig 5:**
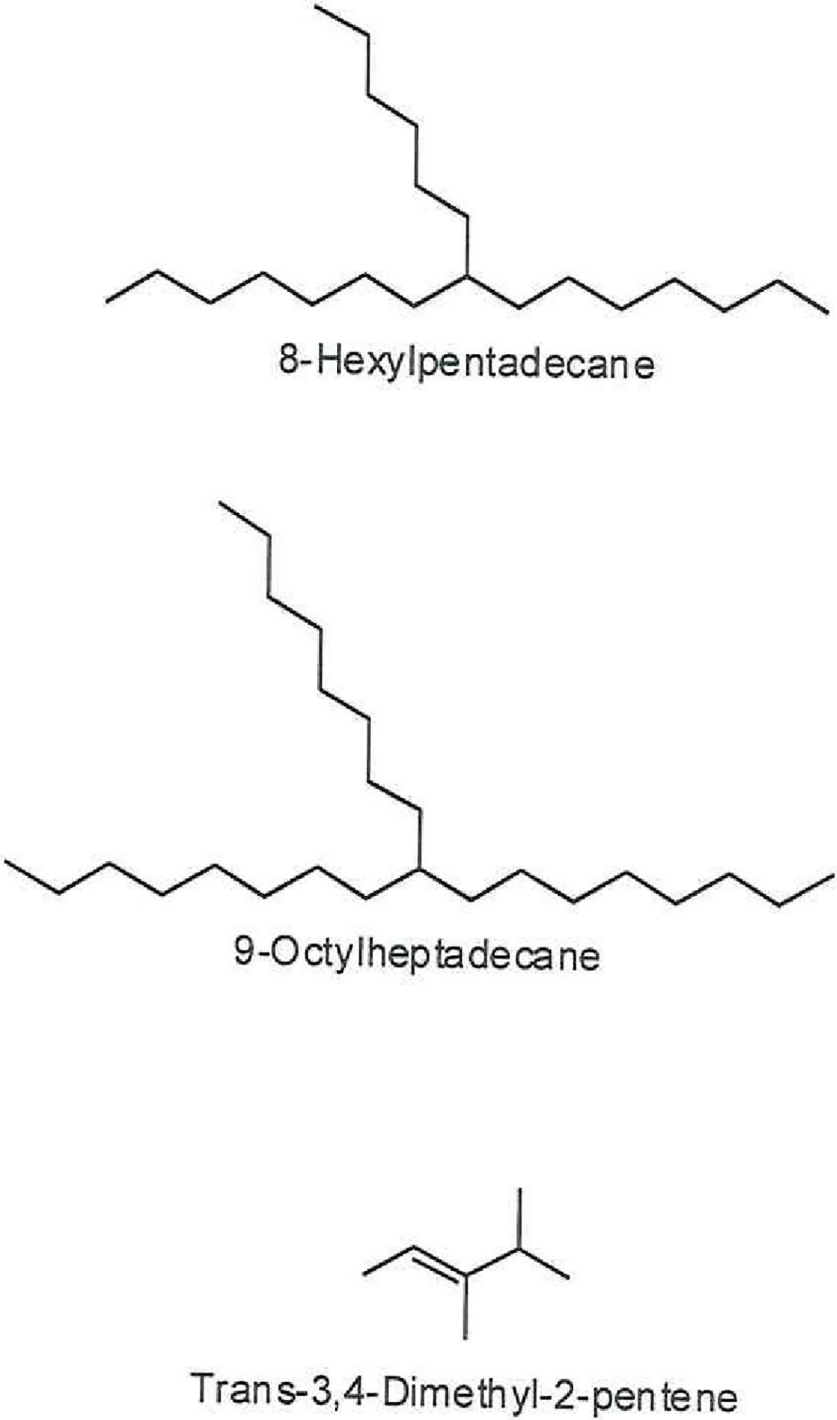
Chemical structures of the major components in the *n*-hexane *C*. *palaestina* extract. 8-Hexylpentadecane is the principal component (54.65%).

In examining the phytochemical composition, GC-MS methodology identified 18 components in the methanol extract of the natural *C*. *palaestina* plant for the first time. Understandably, fewer compounds were identified in the hexane extract. Figs 2 and 3 show the entire ion chromatograms of the methanol and hexane extract injections, respectively. Good resolution was obtained in both chromatograms, since a 79-min analysis scan run was performed, ending with a high temperature of 320°C, to facilitate the elution of high molecular weight compounds out of the capillary GC HP-5 column. Dodecanoic acid isooctyl ester (20.96%), palmitic acid (10.58%), 2-fluoro-3-trifluoromethylbenzoic acid, heptadecyl ester (6.7%), and 2-fluoro-5-trifluoromethylbenzoic acid, heptadecyl ester (6.88%) are the major compounds in the methanol extract. The major chemicals in the hexane extract are 8-hexylpentadecane (54.66%), 9-octylheptadecane (9.33%), and trans-3,4-dimethyl-2-pentene (8.42%). The main components, along with their retention times (RTs) and peak area percentages, are presented in Tables 1 and 2. Figs 4 and 5 show the chemical structures of the major components in the methanolic and hexane extracts, respectively.

**Table 1:** Components of *C*. *palaestina* methanolic extract verified by GC-MS. Three phytochemicals–namely Sesamin, campesterol, and stigmasterol–are bolded and could be the source of the methanolic extract’s anticancer activity.

No.	Compound name	RT (min)	Peak Area %
1	Propanoic acid	11.631	2.619
2	2-Methylbenzaldehyde	17.653	5.376
3	1-Formylpyrrolidine	19.460	1.754
4	2-Methoxy-4-vinylphenol	20.298	4.930
5	1,6-Anhydro-β-D-glucose	24.872	1.543
6	Hexanoic acid	27.714	1.087
7	Dodecanoic acid isooctyl ester	35.149	20.965
8	Palmitic acid	35.273	10.582
9	(Z,Z)-9,12-Octadecadienoic acid	38.382	1.755
10	Palmitoleic acid	38.503	3.915
11	Oleic acid	38.800	1.860
12	2-Fluoro-3-trifluoromethylbenzoic acid, heptadecyl ester	45.469	6.700
13	2-Fluoro-5-trifluoromethylbenzoic acid, heptadecyl ester	46.236	6.875
14	**Sesamin**	**53.502**	**4.490**
15	ethanone, 2-(3H-imidazo[4,5-b]pyridin-2-ylthio)-1-(4-morpholinyl)-	54.172	3.222
16	Campesterol	54.359	1.449
17	Stigmasterol	54.689	1.007
18	Pregn-5-en-3-ol, 21-bromo-20-methyl-, (3.beta.)-	55.429	4.461

**Table 2:** Components of *C*. *palaestina* hexane extract as determined by GC-MS. Sesamin is bolded and could be the source of the hexane extract’s anticancer activity.

No.	Compound name	RT (min)	Peak Area %
1	Trans-3,4-Dimethyl-2-pentene	9.401	8.418
2	Eicosane	44.622	0.654
3	Heneicosane	47.610	1.782
4	Docosane	49.023	0.98
5	8-Hexylpentadecane	50.409	54.656
6	11-Butyl-docosane	51.709	1.735
7	9-Octylheptadecane	52.998	9.326
8	**Sesamin**	**53.505**	**1.138**
9	2-(1-adamantyl)ethyl 2-phenylacetate	55.428	2.112

**Fig 6:**
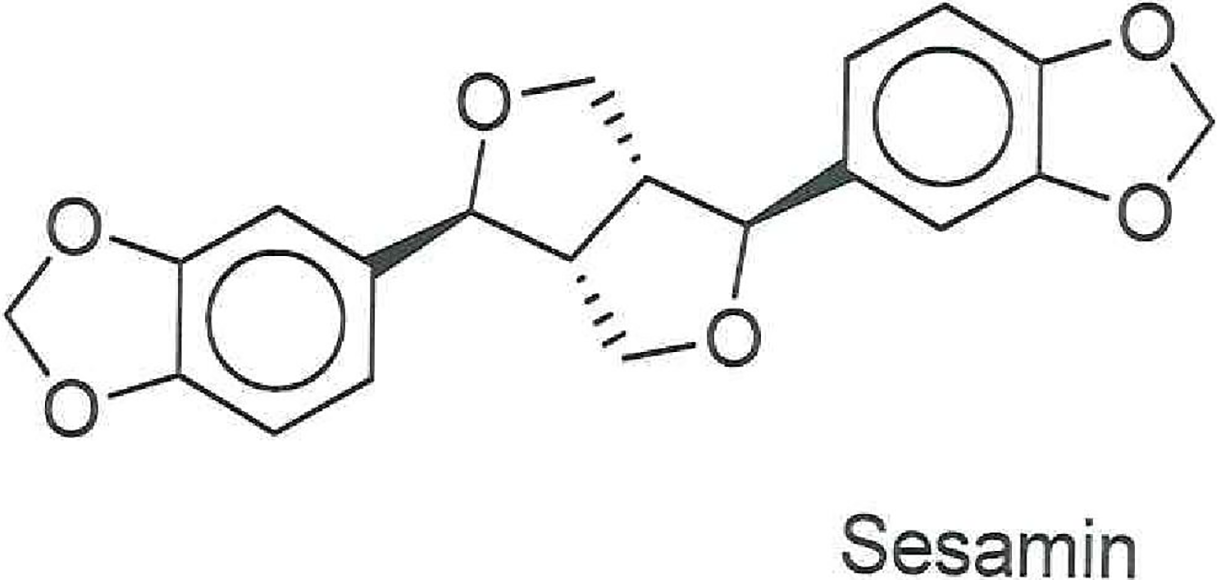
Chemical structure of Sesamin, the phytochemical potentially responsible for anticancer activity in the *C*. *palaestina* hexane extract and the major source of the methanolic extract activity.

**Fig 7:**
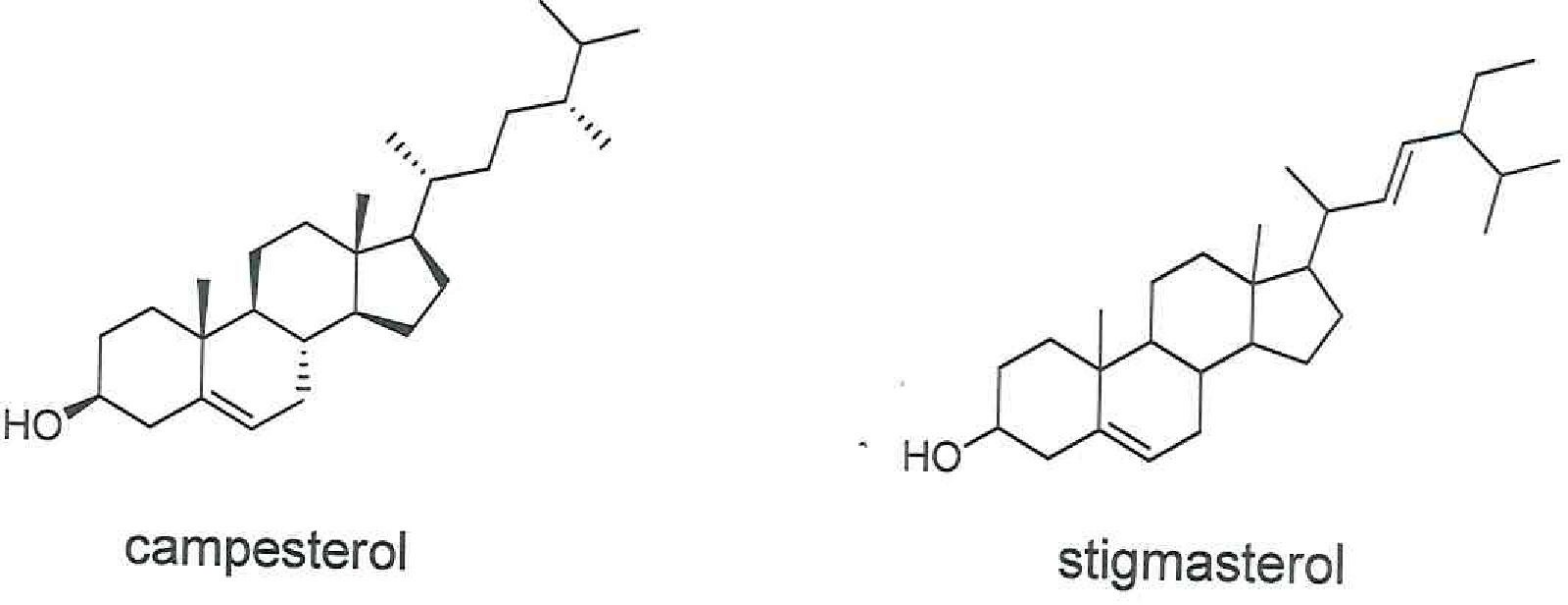
Chemical structures of Campesterol and Stigmasterol, phytochemicals found in the methanolic extract of *C*. *palaestina*.

Sesamin (see Fig 6), which exists in the oil of sesame seeds and some other plants, was one of the major components, as shown in Table 1. It exhibits a variety of biological activities, such as lipid-lowering [13], antihypertensive [14], antioxidant [15], and anticancer effects. [16] In regard to its antitumor effects, Sesamin has already been confirmed to be active against several cancer cell types, including prostate cancer, breast cancer, colon cancer, and human lung cancer[17–19]. Other phytochemicals (see Fig 7), which belong to the phytosterols—namely Campesterol (1.5%) and Stigmasterol (1.0%)—were also noticed, but to a lesser extent, Phytosterols are structurally similar to cholesterol and are validated in their anti-carcinogenic effects. For example, Campesterol has been shown to act as biomarker for cancer prevention and is reported to have potential antiangiogenic action via an inhibition of endothelial cell proliferation and capillary differentiation. [20] Moreover, Stigmasterol is reported to significantly inhibit tumor promotion in two-stage carcinogenesis in mice. [21] As well, Dodecanoic acid isooctyl ester and palmitic acid are the dominant compounds, demonstrating 20.9% and 10.58% decrease in tumor size, respectively.

The hexane extract, on the other hand, was tested for the sake of comparison with the polar methanol extract. It identified fewer compounds, mainly hydrocarbons, among which 8-hexylpentadecane was the principal compound (54.65%). Sesamin was present, but in lower concentrations than in the methanol extract. The other two phytosterols (Campesterol and Stigmasterol) were not found in the hexane extract.

### Quantitation of extracted Sesamin using different solvents by HPLC-PDA

**Fig 8:**
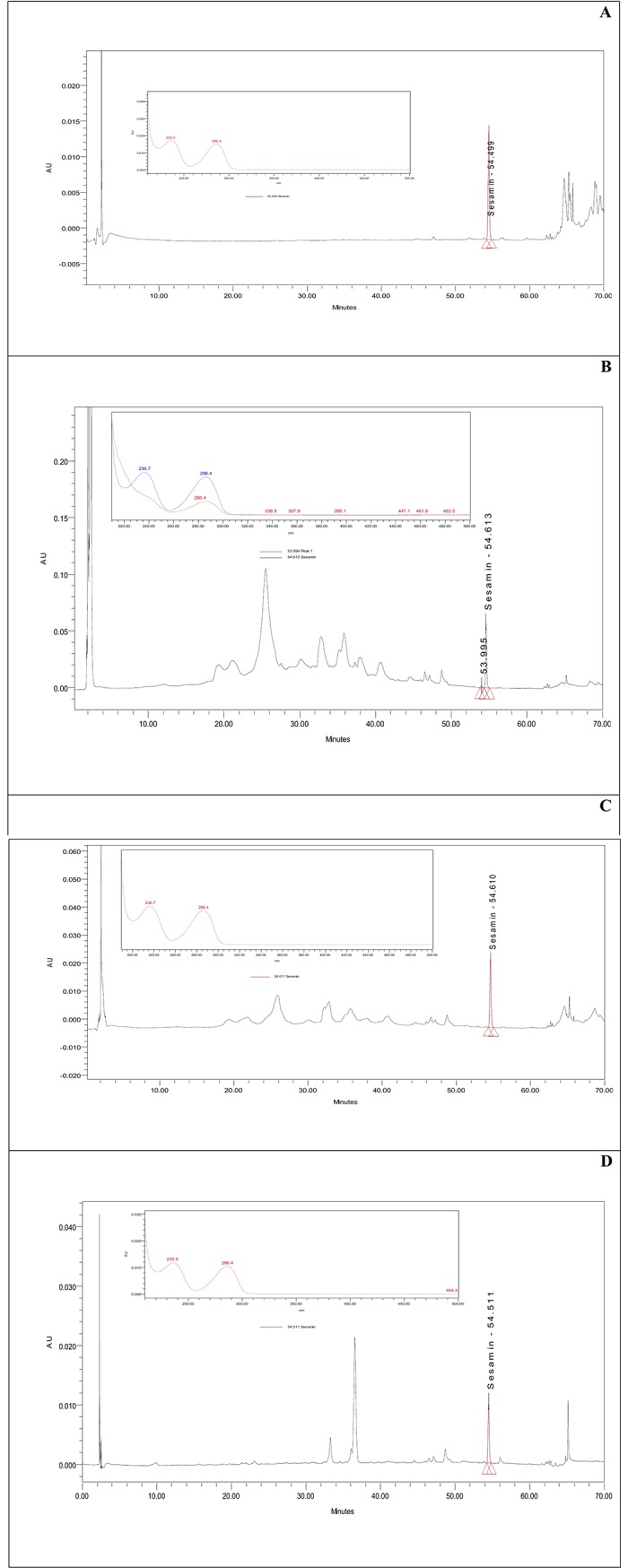
**Typical analytical HPLC-PDA chromatogram of the Sesamin peak and its relevant UV-Vis spectrum in extracts of (A) hexane, (B) methanol, (C) ethanol, and (D) chloroform**.

Sesamin was extracted from *C*. *palaestina* with the use of different solvents under identical experimental conditions, followed by injection into the HPLC. Retention times and the Sesamin stored standard UV-Vis spectrum were used to confirm the identity and specificity of the extracted Sesamin. Aqueous extract showed a negligible amount of Sesamin. Fig 8 portrays the chromatographic profile and the corresponding UV-Vis spectra of the Sesamin extracted from hexane, methanol, ethanol, and chloroform. Although all the chromatograms were recorded at the maximum wavelength (285 nm) to quantify Sesamin, many other peaks were seen preceding and succeeding Sesamin in the extracts. In the PDA stored UV-Vis spectra, the matching of the peaks in the A, B, C, and D extracts with the standard Sesamin peak indicates high purity and specificity, and, therefore, the feasibility of utilizing preparative HPLC for scaling-up purposes in future investigations. Methanol contained the greatest amount of Sesamin (21.7 ppm) in comparison to the other solvents, and hexane the least (6.1 ppm) (Table 3).

**Table 3:** Sesamin concentrations and percentages in different solvents.

Extract	RT (min)	Area (ppm)	Concentration (ppm)	% Sesamin
Hexane	54.499	175598	6.1	0.68
Methanol	54.613	625860	21.7	0.22
Ethanol	54.610	308191	10.7	0.43
Chloroform	54.511	116387	9.168	0.53

### Is Sesamin produced in *C*. *palaestina* or acquired from host plants?

**Fig 9:**
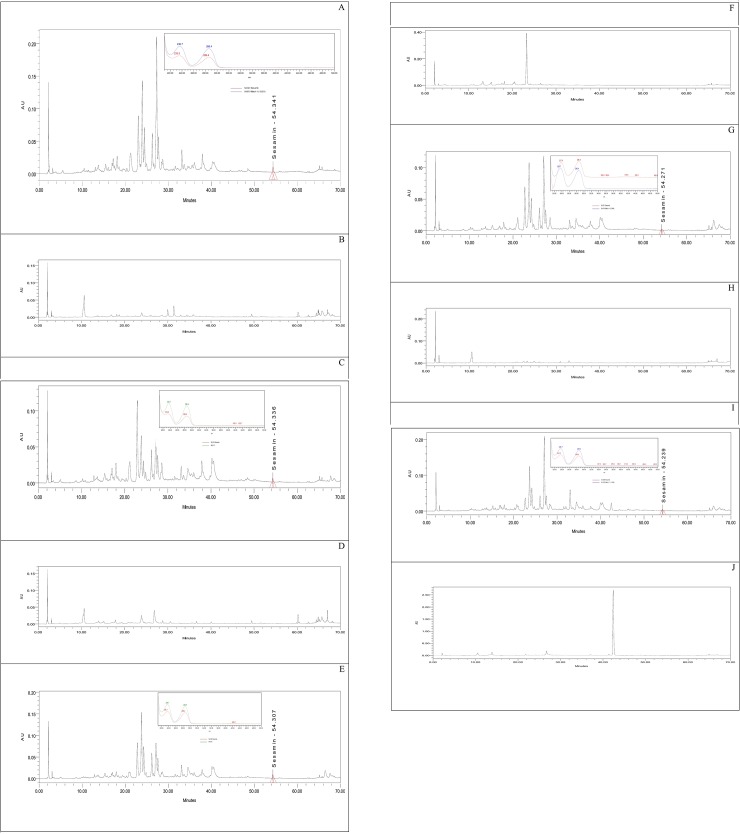
(A) Chromatogram and UV-Vis spectra of extracted *C*. *palaestina* that is grown on *Malva sylvestris*, (B) chromatogram *Malva sylvestris*, (C) chromatogram and spectrum of *C*. *palaestina* that is grown on *Cichorium intybus*, (D) chromatogram of *Cichorium intybus*, (E) chromatogram and spectrum of *C*. *palaestina* that is grown on *Prosopis farcta*, (F) chromatogram of *Prosopis farcta*, (G) chromatogram and spectrum of *C*. *palaestina* that is grown on *Portulaca oleracea*, (H) chromatogram of *Portulaca oleracea*, (I) chromatogram and spectrum of *C*. *palaestina* that is grown on *Corchorus olitorius*, (J) chromatogram of *Corchorus olitorius*.

To verify whether the Sesamin is endogenous secondary metabolite to *C*. *palaestina* or originates merely from the host plant, different samples from the host plants alone, along with *C*. *palaestina* alone, were extracted, and the Sesamin concentration was calculated (shown in Fig 9). The five host plants were *Malva sylvestris*, *Cichorium intybus*, *Prosopis farcta*, *Portulaca oleracea*, and *Corchorus olitorius*. Table 4 shows the Sesamin concentrations in methanolic extracts of *C*. *palaestina* that were parasitic to the aforementioned plants. Since the Sesamin peaks were not seen in the chromatograms of the host plants, it was concluded that Sesamin is endogenous to *C*. *palaestina*.

**Table 4:** Sesamin concentrations in *C*. *palaestina* (CP) that are parasitic on other plants (termed host plants).

Sample CP that is grown on host plant	RT (min)	Area	Concentration (ppm)
CP that is grown on *Malva sylvestris*	54.341	86256	8.174
CP that is grown on *Cichorium intybus*	54.336	59728	7.301
CP that is grown on *Prosopis farcta*	54.307	94565	8.448
CP that is grown on *Portulaca oleracea*	54.271	21222	6.033
CP that is grown on *Corchorus olitorius*	54.239	40460	6.666

## Conclusion

Sesamin is well documented in the scientific literature as a lipid-lowering agent, an antihypertensive, antioxidant, and anti-cancer drug candidate. It is one of the principal lignan secondary metabolites that are commonly isolated from sesame seeds. However, the results of the current study show that natural *C*. *palaestina* contains a sufficient amount of Sesamin, about 0.68%, when methanol as used as the extracting solvent. Following the determination of the Sesamin content in *C*. *palaestina*, we raised the question: Is Sesamin produced in *C*. *palaestina* or acquired from the host plant? The quantitation of the Sesamin content in five host plants and a comparison to the content in *C*. *palaestina* revealed that Sesamin is an endogenous metabolite in *C*. *palaestina*. Further study is required to verify whether *C*. *palaestina* could be a valuable source for the production of Sesamin or other anti-cancer phytochemicals, such as campesterol and stigmasterol. As well, we are wondering if genes/ proteins in the Sesamin production pathway could be valid biological targets for the development of novel and selective pesticides for *C*. *palaestina* and other *Cuscuta* species. A big question is raised of whether inferring Sesamin production in *C*. *palaestina* could reduce its attack ability to host plants.
